# Effect of Low Power Laser on Incisional Wound Healing

**Published:** 2007-01-20

**Authors:** Masoud Parirokh, Shahriar Dabiri, AliReza Bahrampour, Mahmoud Homayon zadeh, Mohammad Jafar Eghbal

**Affiliations:** 1 *Department of Endodontics, Dental School, Kerman University of Medical Sciences, Kerman, Iran*; 2 *Department of Pathology, Medical School, Kerman University of Medical Sciences, Kerman, Iran*; 3 *Department of Mathematics, Vali-Asr Rafsanjan University of Medical Sciences, Rafsanjan, Iran*; 4 *General Practitioner, Rafsanjan, Iran*; 5 *Department of Endodontics, Dental Research Center, Shahid Beheshti University of Medical Sciences, Tehran, Iran*

**Keywords:** HeNe laser, Healing, Incisional wound, Low power laser

## Abstract

**Introduction: **The effectiveness of low power lasers for incisional wound healing, because of conflicting results of previous research studies, is uncertain. Therefore, this study was carried out to evaluate low power laser effects on incisional wound healing.

**Materials and Methods: **Incisional wound was produced on thirty-six mature male guinea pigs under general and local anesthesia. In half of the cases, HeNe laser radiations were used for five minutes and the rest were left untreated Animals were divided into six groups of six animals each that were killed after 3, 5 and 14 days. After histopathology processing and H&E staining, specimens were examined for acute and chronic inflammations, epithelial cell migration, epithelial seal and barrier formation, fibroblast migration, fibrosis, clot formation and granulation tissue formation. Mann-Whitney U and the Wilcoxon tests were used for statistical analysis.

**Results: **Statistically significant differences were found between fibroblast migration, acute and chronic inflammation of radiated groups and the control group at 5 days interval (p<0.05). There was no statistically significant difference at 3 and 14 days between laser radiated and control groups.

**Conclusion: **This study showed that HeNe laser had beneficial effects on incisional wound healing particularly at 5 days interval; however, further research on chronic ulcers is recommended.

## Introduction

LASER is an acronym for light amplification by stimulated emission of radiation and has been used in medical sciences since 1960s. Laser in dentistry is often associated with high power lasers that burn or disintegrate tissues (1,2). Little has been published about the use of low power lasers in dental practice. Initially, Mester *et al. *published a report on the beneficial effect of this type of laser (3). Low power lasers do not affect tissue thermally but act to increase the rate of repair of injured tissue (4). Studies have shown that low power lasers can affect the biological functions of macrophages (5), angiogenesis (6) and Low power lasers such as Helium-Neon (HeNe), Ruby, Gallium-Aluminum-Asnium (Ga-Al-As) has been reported to have beneficial effects on tissue wound healing in animals as well as in human tissue culture (7,8).

Laser therapy could be useful as a treatment modality in myofascial pain syndrome because of its noninvasiveness, ease, and short-term application (9). Also it was reported to reduce post extraction pain and swelling and to increase rates of wound healing (2). However, some studies in which red spectrum laser were used resulted in confusing data and conflicting findings. Some of these studies indicated that the biostimulation effect did not occur in all but some cases of laser irradiation (2,7,8,10). Few controlled studies were carried out in order to identify the beneficial effects of HeNe laser bio-stimulation. Ethical concern, bulky equipment and difficulties with sound study design have precluded a precise evaluation of laser bio-stimulation (11). Most of earlier studies on oral tissues were observational (12), or clinical data collection on pain, swelling and discomfort (2,13,14). Therefore, the purpose of this study was to determine the histopathological effect of the HeNe laser on oral surgical wound healing.

## Materials and Methods

The research protocol was approved by the Ethics committee of Kerman University of medical sciences. The study comprised of 36 adult male guinea pigs with approximate weight of 400-450g. The animals were given intraperitoneal injection of 7.5 mg.kg-' Ketamin HCl (Alfasan, Woerden, Netherlands) and 0.1 mg.kg-' Xylazine (Bayer, Munich, Germany). After anesthesia, the head and neck of the animals were scrubbed with betadine iodine (Daropakhsh, Tehran, Iran). An infiltration injection of 2% lidocaine with 1:80000 epinephrine (Daropakhsh, Tehran, Iran) was then made.

In each animal a triangular incision was made in the anterior portion of the mandible. Having reflected the flap for five minutes, it was then sutured by #4/0 silk (Supa, Tehran, Iran). Experimental groups constituted half of the animals received HeNe laser radiation (LR) (Nuclear energy organization, Tehran, Iran) for lmin The wave length of HeNe laser was about 632.8 nm with 5 mW output and the zone of radiation had 2 mm diameter. It could produce 2.5 J/Cm^2^ energy in the tissues. The rest of animals received no radiations. Animals were divided into 6 groups of 6 each which comprised of 3 radiated and 3 controls.

They were then killed by intracardiac injection of Ketamin and Xylazine after 3, 5 and 14 days. Anterior portion of the mandible was removed as a block section and send in 10% formalin for pathologic examination. After histopathologic processing and H&E staining, the specimens were evaluated according to Harrison and Jurosky (15) by unawareness of time intervals and laser treatment for clot formation, acute and chronic inflammation, epithelial cell migration, epithelial seal formation, epithelial barrier formation, fibroblast migration, fibrosis and granulation tissue formation.

The Mann-Whitney U and the Wilcoxon tests were used for analysis of data relating to histopathologic results.

## Results

Two laser and one control specimens at 3 days interval were excluded because of processing problem. Histopathologic results of the remaining specimens were as follows:

3-Days interval: All specimens in both control and laser irradiated groups showed epithelial migration and crust between two edges of surgical incision area. Epithelial seal could be observed in one of the laser radiated (LR) specimens. In the rest of specimens the epithelial seal and barrier did not form. Polymorphonuclears, macrophages and plasma cells were observed in both control and experimental groups with no significant differences.

**Figure 1:**
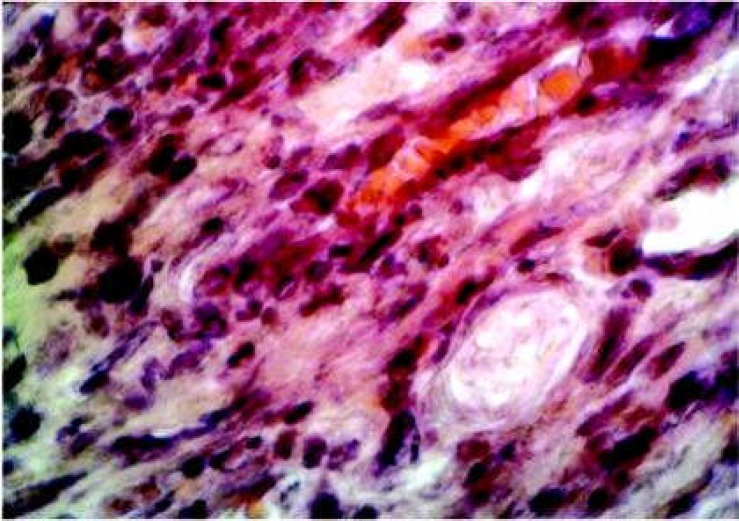
Inflammatory cells in 5-day non radiated group showing smaller fibroblast and more inflammatory cells (x20).

**Figure 2:**
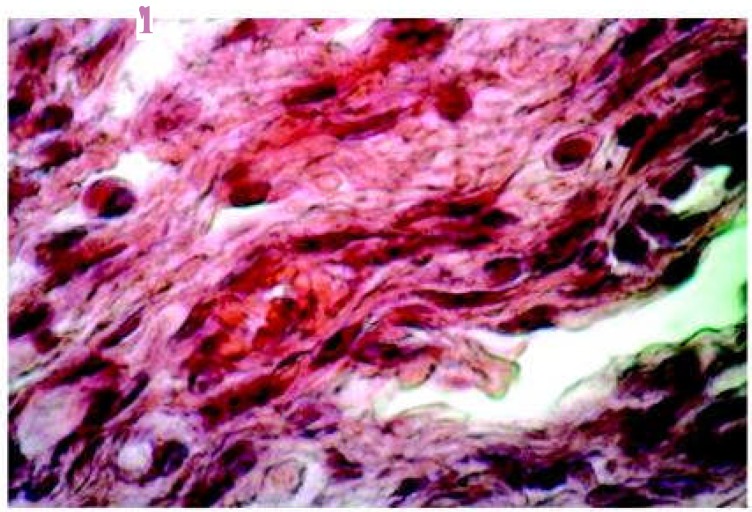
Five-days radiated group showing decreasing number of inflammatory cells and enlarged fibroblasts compared with smaller size of non-radiated group (x20).

**Figure 3:**
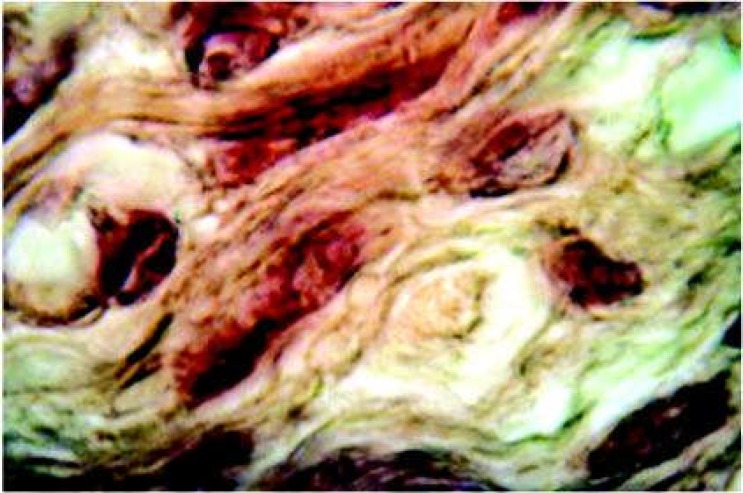
Higher magnification of plumped fibroblast (x40).

5-Days interval: Significant differences were observed between 3 and 5 days in both LR and control groups. Healing in all 5^th^ day specimens was better than 3^rd^ day animals. There was significant differences between LR and control group in relation to the fibroblast migration, acute and chronic inflammation, clot formation and fibrosis (p<0.05) (Table1). The number of inflammatory cells in LR group was lower than the control group (Figures 1, 2). Plumped fibroblasts (Figure 3) were very evident in the LR specimens (p<0.05). Although specimens of control group showed more tissue maturation than radiated group, there were no statistical differences between epithelial seal and barrier formation between LR and control groups (p>0.05).

14-Days interval: There was no significant difference between LR and control groups at 14 days. Epithelial barrier was completed and inflammation and fibrosis were similar in both groups.

## Discussion

Promotion of healing is of paramount importance in medicine, particularly in diabetic and immuno-compromised patients (16). There have been various studies performed on low power laser; however, conflicting results and few oral researches motivated the researchers to conduct this study. In this study, HeNe laser was used and the results showed that in the LR group particularly at 5 days interval, healing was more evident than non radiated group. This was similar to the results of some previous studies (13,17-19) although it was in conflict with the results of many other investigations (8,10,11,14). Researchers of previous studies believed that the differences between fluency-energy level in tissues (2,4,13) , frequency of radiation (12), systemic effect (17,20,21) and the type of ulcer (19) would influence the results of low power laser exposures and

produced conflicting results. It is believed that the optimum tissue-healing rates at HeNe laser exposure levels exist between I J/Cm2 -20J/Cm^2^ (2). This amount of energy could induce metabolic changes within the cells. In this study, the energy level produced in tissue was 2.5 J/cm^2^. Results showed that fibroblast proliferation was significantly more evident in the LR than control group in 5 days interval which was in agreement with previous studies in which low power laser beneficial effects were demonstrated (2,18,19).

Studies of Mester *et al. *and Abergel *et al. *showed that the frequency of radiation could improve tissue healing rates (3,18). However, in this study, despite a single radiation exposure significant differences were found between LR and control groups in inflammation and fibroblast migration at 5-day intervals. Neiburger and Yu *et al. *showed the same finding after single laser radiation (2,4). Funk et al. showed those 30 minutes after laser radiation of peripheral mononuclear blood cells, IL 1 a, IL2, TNFa and INFT increased significantly (7). It might be one of the reasons that even with one radiation exposure, the beneficial effects of HeNe laser could be observed in the present study.

The systemic effect of these cytokines was confirmed by Belkin and Schwartz as well as Karu and Inoue (17,19,20). Therefore, in many studies, as both laser radiation and control procedure were performed on the same patient, the laser radiation would not produce precise results (8,14,21). This is the reason for using different guinea pigs for control and LR groups in the present investigation.

The type of ulcer could affect radiation response. Many researchers believed that old ulcers, because of low oxygen concentration, PH and nutrients showed a better response to low power laser than fresh ulcer (19). This study, as well as others, demonstrated that HeNe laser has beneficial effects on fresh ulcers (2,18,22,23).

## Conclusion

In conclusion this study showed that HeNe laser has had beneficial effects on incisional wound healing particularly at 5-days interval. However, further research on chronic ulcers is recommended.

## Footnote

**On the page 46, in the** *IEJ* **number 2 the Methods and Materials of this article was missed, so the article is presented again.**
